# Clinical sensitivity and specificity of multiple T2-hyperintensities on brain magnetic resonance imaging in diagnosis of neurofibromatosis type 1 in children: diagnostic accuracy study

**DOI:** 10.3325/cmj.2011.52.488

**Published:** 2011-08

**Authors:** Zlatko Sabol, Biserka Rešić, Romana Gjergja Juraški, Filip Sabol, Matilda Kovač Šižgorić, Krešimir Oršolić, David Ozretić, Dubravka Šepić-Grahovac

**Affiliations:** 1 Sabol Outpatient Clinic for Sick Children, Zagreb, Croatia; 2 Department of Pediatrics, University Hospital Zagreb, Clinical Medical Center, Zagreb, Croatia; 3 Department of Pediatrics, University Hospital Split, Split, Croatia; 4 Department of Pediatrics, Sisters of Mercy University Hospital, Zagreb, Croatia; 5 Department of Radiology, Holy Ghost University Hospital, Zagreb, Croatia; 6 Clinical Institute of Diagnostic and Interventional Radiology, University Hospital Zagreb, Clinical Medical Center, Zagreb, Croatia; 7 Department of Neurology, Clinical Hospital Center Rijeka, Rijeka, Croatia

## Abstract

**Aim:**

To determine the prevalence, number, and location of multiple (≥2) T2-hyperintensities on brain magnetic resonance imaging (MRI) in children with neurofibromatosis type 1 (NF1) and their correlation with age, and to establish their sensitivity, specificity, and accuracy for the diagnosis of NF1 in children, especially in the early age (2-7 years).

**Methods:**

We performed a cross-sectional study of 162 patients with NF1 from Croatian Neurofibromatosis Association Database and 163 control children between the ages of 2 and 18 years who underwent brain MRI between 1989 and 2009.

**Results:**

Multiple T2-hyperintensities were present in 74% of NF1 patients and 1.8% of controls. They were mainly located in the basal ganglia, brainstem, and cerebellum and were significantly decreased in prevalence and number in the older age. T2-hyperintensities had excellent diagnostic accuracy with the area under the receiver operating characteristic (ROC) curve of 0.849 and 95% confidence interval (CI) of 0.805-0.886. The diagnostic sensitivity, specificity, and accuracy rate of T2-hyperintensities for NF1 were highest in the youngest age (2-7 years): 81% (95% CI 71%-89.1%), 99% (95% CI 92.3%-100%), and 85.8 (95% CI 83.3-93.8), respectively.

**Conclusion:**

This study strongly suggests the inclusion of T2-hyperintensities on brain MRI on the list of diagnostic criteria for NF1, especially in children of early age, when the clinical penetration of the *NF1* gene has not yet been completely finished.

Neurofibromatosis type 1 (NF1) or von Recklinghausen's disease is one of the most common autosomal dominant inherited diseases in humans with an estimated birth incidence of 1/2500 and a disease prevalence of 1/3000-4000 (1). *NF1* gene is located on the pericentromeric region of chromosome 17q11.2 (2). It is a megagene, spaning 350 kb of genomic DNA and consisting of 60 exons encoding an 11-13 kb GTPase activating protein – neurofibromin of 2818 amino acids (3,4). The mutation rate for *NF1* gene is high. A half of all NF1 cases are familial, while the other half is caused by a new mutation (5). Genetic molecular testing confirms the disease’s existence but has no predictive value for its severity and course.

In the past thirty years, the diagnosis of NF1 was made using a set of clinical criteria developed by the National Institutes of Health Consensus Conference, so called NIH diagnostic criteria (6). The diagnosis of NF1 is based on the presence of two or more of the following: 1) six or more *café au lait* macules, the greatest diameter of which is more than 0.5 cm in prepubertal patients and more than 1.5 cm in postpubertal patients; 2) two or more neurofibromas of any type, or one plexiform neurofibroma; 3) freckling in the axillary or inguinal region; 4) optic glioma; 5) two or more Lisch nodules; 6) a distinctive osseous lesion such as sphenoid dysplasia or pseudarthrosis; 7) a first-degree relative with NF1 according to the preceding criteria. However, the diagnosis cannot always be made in all children using the above mentioned criteria, especially in early childhood when the penetration of *NF1* gene in usually not complete (7,8).

The cranial magnetic resonance imaging (MRI) is the best method for showing many features of NF1, including optic pathway gliomas, brain tumors of various locations, brain stem tumors, and orbital neurofibromas. Most frequent brain changes in children with NF1 are areas of increased T2-weighted signal intensity – T2-hyperintensities or “unidentified bright objects,” as they usually cannot be visualized using T1-weighted imaging. T2-hyperintensities are age-related findings on MRI and have been observed in 43%-93% of children who suffer from NF1 (9-11). These lesions do not exert mass effect, contrast enhancement, or surrounding edema. They are most commonly found in the basal ganglia, thalamus, cerebellum, and brainstem (12). Former research on multiple T2-hyperintensities on brain MRI in children as diagnostic criterion for NF1 was contradictory and scarce (13-17).

The aim of this cross-sectional study was to determine the prevalence, number, and location of multiple (≥2) brain T2-hyperintensities on MRI, and their correlation with age in children with NF1. A further aim was to determine the indicators of diagnostic accuracy of T2-hyperintensities in children of different ages, especially in the early age (from 2 to 7 years) when the *NF1* gene penetration is still not completed.

## Patients and methods

### Study design, setting, data collection, and patients

This cross-sectional study with prospective data collection was performed at Sabol Outpatient Clinic for Sick Children in Zagreb, Croatia, from March to November 2010. It was approved by the Ethics Committee of Zagreb University School of Medicine.

The study included 325 patients of both sexes between 2 and 18 years old, divided into two groups. The NF1 patient group consisted of 162 children who were diagnosed with NF1 by the NIH diagnostic criteria (6) and underwent cranial MRI examinations. They were selected from the Croatian Neurofibromatosis Association Database, created by the main investigator on the basis of routine clinical follow-up of NF1 patients between January 1989 and December 2009 at the Department of Pediatrics, University Hospital Zagreb, Clinical Medical Center, and Sabol Outpatient Clinic for Sick Children in Zagreb. For each patient, data were available about NIH diagnostic criteria, presence or absence of T2-hyperintensities on cranial MRI examination, their number and location, as well as data on other changes and complications of the disease. Although some of the patients had more than one MRI scanning, cross-sectional analysis included only the data about clinical NIH diagnostic manifestations of NF1 and MRI changes at the time of the first MRI.

The control group included 163 patients selected by accidental sampling from patient database of the above mentioned institutions (3542 patients) during the same period as the patient group who performed the brain MRI examination for other complaints not related to NF1. The exclusion criteria for control group were diseases in which T2-hyperintensities were expected: hypoxic-ischemic encephalopathy, intracranial hemorrhage, infection, changes caused by radio- and/or chemotherapy, neurometabolic diseases, brain heredodegenerative diseases, tumors, multiple sclerosis, and acute disseminated encephalomyelitis. NF1 and other neurocutaneous diseases were excluded by careful clinical examination. All patients of both groups were clinically examined and prospectively evaluated by the head investigator.

### Brain MRI

The examinations of participants of both groups were done with the MRI scanners operated at different magnetic field strength in different medical institutions in Croatia. The brain MR images were performed with a low- and medium-field systems (0.2-1.0 Tesla) on 90 children from NF1 patient group and 79 children from the control group, and with high-field MRI systems (1.5 -3 Tesla) on 72 children from the NF1 patient group and 84 children from the control group. Standard spin-echo T1- and T2-weighted sequences in the axial and sagittal planes, respectively, and axial fluid-attenuated inversion recovery T1- and T2-weighted sequences were obtained in all patients. Additional T1- or T2-weighted coronal images were obtained in most patients. A contrast agent (gadopentetate dimeglumine) was administered at 0.1 mmol/kg to 91 of 162 (56.2%) children from NF1 group and to 2 of 163 (1.2%) children from the control group. No adverse events were noticed in either of the groups.

All MR scans were examined independently by two neuroradiologists, who used visual inspection and were blinded for each group of patients. T2-hyperintensities on MRI were defined operationally as areas of confluent hyperintensity (signal intensity higher than that of cortical gray matter) on the proton density and the T2-weighted images without associated mass effect. A specific attention was paid to the number of lesions, location, signal characteristics on T1- and T2-weighted images, and presence or absence of contrast enhancement. In case of incompatible findings, the consensus between two neurologists was made.

### Reference standard

The basic reference standard – NIH diagnostic criteria for NF1 (6) were used to determine or exclude a definitive clinical diagnosis of NF1. Presence of at least two of seven NIH diagnostic criteria confirmed clinical diagnosis of NF1 in children.

### Statistical analysis

For continuous variables, such as the number of T2-hyperintensities or age at presentation, the differences between groups were compared using *t* test. Categorical variables such as sex, type of NF1 (sporadic or familial), and brain location of T2-hyperintensities were estimated in terms of relative frequencies. For these variables, the comparison between two groups was made using contingency tables and χ^2^ test. For the analysis of correlation of number of T2-hyperintensities and the age of patients, the Pearson test of correlation was performed. For the analysis of association of prevalence of T2-hyperintensities and the age of patients, children were grouped according to age. For presence/absence of T2-hyperintensities on brain MRI, we constructed a 2 × 2 contingency table that consisted of true-positive, true-negative, false-positive, false-negative, and true-negative results in accordance with the reference standard used in each case. The sensitivity and specificity of T2-hyperintensities as diagnostic criterion for NF1, as well as the negative and positive predictive value, odds ratio (OR), and accuracy rate were calculated by using standard formula. Indicators of diagnostic accuracy were evaluated in different age groups: 2-7 years, 8-12 years, and 13-18 years. The accuracy of T2-hyperintensities in diagnosis of NF1 in children was verified by receiver operating characteristic (ROC) curve. *P* value <0.05 was indicated as significant difference.

## Results

### The clinical and epidemiological characteristics of patients and controls

The patient and control group were comparable according to age and sex. The median age of 162 children in the NF1 patient group (91 boys and 71 girls) at the time when the definite clinical diagnosis of NF1 was made was 5.6 years (range 0.3-17.9). The median age of children with NF1 and of 163 control group children (82 boys and 81 girls) at the time when brain MRI examinations were made was 7.7 years (range 1.9-18.3) and 8.2 years (range 2.0-17.9), respectively (*P* = 0.196). Of the children with NF1, 87 (53.7%) were familial and 73 (45.1%) were sporadic cases. For two adopted children (1.2%), family history was unknown. Ten NF1 patients, all sporadic cases with multiple *café au lait* spots as the only diagnostic sign of NF1, underwent brain MRI examination prior to meeting the NIH diagnostic criteria. At the age of 13, they all met the NIH diagnostic criteria for NF1.

### Prevalence and location of T2-hyperintensities on brain MRI

Multiple (≥2) T2-hyperintensities on brain MRI were found in 119 of 162 children with NF1 (73.5%) and only 7 of 163 children in the control group (4.3%) (*P* < 0.001). Four children in the control group had a single lesion and other 3 had two or more lesions. The actual prevalence of multiple T2-hyperintensities in the control group was 1.8% (3/163 patients).

**Figure 1:**
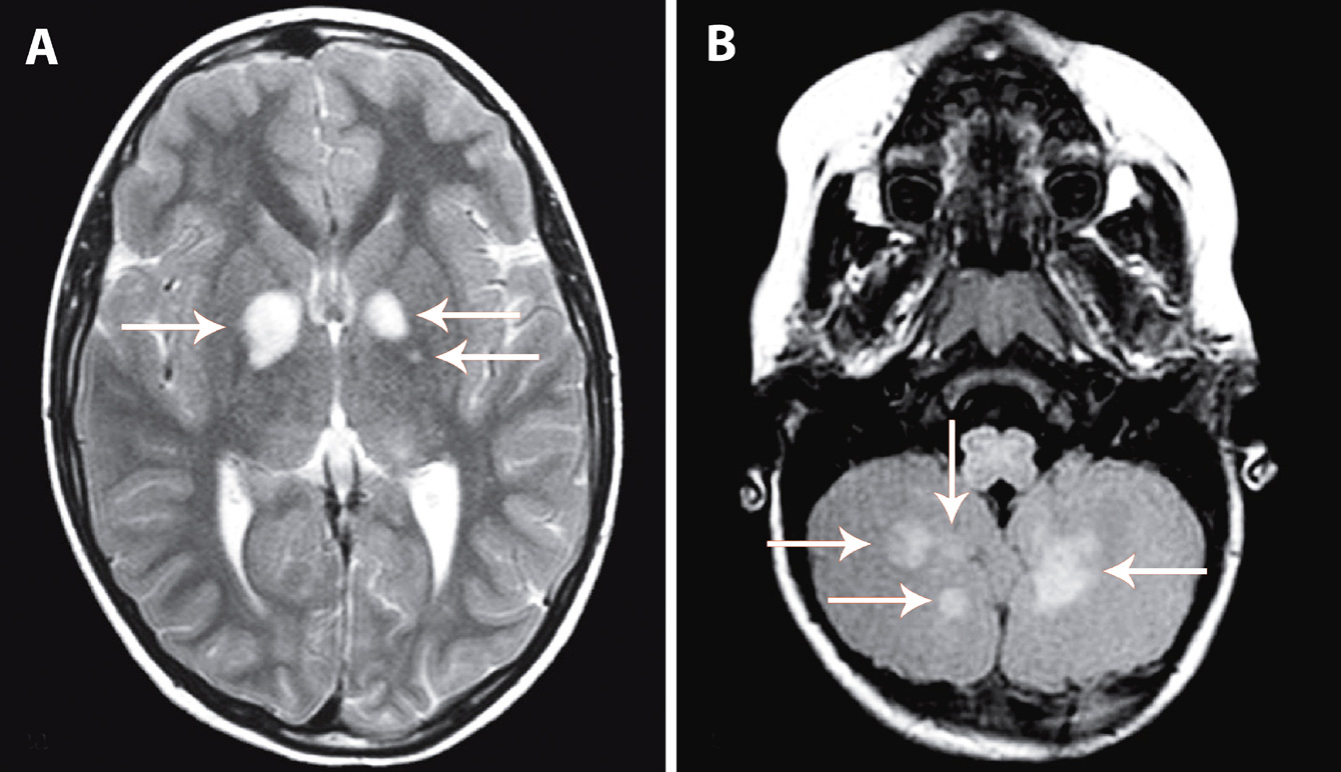
Multiple hyperintensities on T2-weighted cranial magnetic resonance imaging in children with neurofibromatosis type 1. Axial image shows multiple oval discrete lesions with increased intensity (arrows) in the globus pallidus (**A**); axial fluid attenuated inversion recovery image shows multiple diffuse T2-hyperintensities (arrows) in the cerebellum (**B**).

In the NF1 group, there were 636 T2-hyperintesities (median 3, range 0-16) and in the control group there were only 11 (median 0, range 0-3) (*P* < 0.001). T2-hyperintensities in the patient group were mainly located in the basal ganglia (81%), most frequently in the globus pallidus (78%), brainstem (38%), and cerebellum (36%) (Figure 1A and 1B). In the control group, they were located in the subcortical structures of the cerebrum (3/7 patients) and parasagittal watershed regions (3/7 patients). Only one patient in the control group had two T2-hyperintensities in the basal ganglia (putamen) (Table 1). The overall agreement in identification of multiple (≥2) T2-hyperintensities between the two radiologists was 99.4%. The agreement for localization and total number of lesions was 91.4% and 77%, respectively.

**Table 1:** Prevalence, total number, and locations of T2-hyperintensities on brain magnetic resonance imaging in children with neurofibromatosis type 1 (NF1) (n = 162) and children without NF1 (n = 163)

	Children				T2-hyperintensities in children			
	with NF1		without NF1		with NF1		without NF1	
T2-hyperintensities	No.	%	No	%	No.	median (range)	No.	median (range)
Prevalence	119/162*	73.5	7/163*	4.3	636^†^	3 (0-16)	11^†^	0 (0-3)
Location of lesions:								
basal ganglia	96/119	80.7	1/7	14.3		1 (0-9)		0 (0-2)
globus pallidus	93/119	78.2				1 (0-7)		
nucleus caudatus	19/119	16.0				0 (0-3)		
putamen	17/119	14.3	1/7	14.4		0 (0-3)		0 (0-2)
brainstem	45/119	37.8				0 (0-6)		
cerebellum	43/119	36.1				0 (0-7)		
cerebrum	31/119	26.0	3/7	42.9		0 (0-5)		0 (0-1)
periventricular	0	0	3/7	42.9		-		0 (0-3)

*P < 0.001 vs prevalence of T2-hyperintensities in children with NF1 and children without NF1 (*t* test).

†*P* < 0.001 vs total number of T2-hyperintensities in children with NF1 and children without NF1(*t* test).

There were no significant differences between the groups of patients who were examined with low-, medium-, and high-field MRI systems in the prevalence (72% and 75%, *P* = 0.363) and the number of T2-hyperintensities (median 4, range 1-16 and median 5, range 1-14, *P* = 0.076). Also, there were no significant differences in locations of T2-hyperintensities between two groups: the basal ganglia (*P* = 0.160), brainstem (*P* = 0.554), and cerebellum (*P* = 0.738) (Table 2).

**Table 2:** Prevalence, total number, and locations of T2-hyperintensities on brain magnetic resonance imaging (MRI) in children with neurofibromatosis type 1 (NF1) assessed by low- and medium field MRI (n = 90) and high-field MRI (n = 72).

T2-hyperintensities	No. (%) of children with NF1 assessed by		Median (range) of the number of T2-hyperintensities in children with NF1 assessed by		
	low- and medium-field MRI	high-field MRI	low- and medium-field MRI	high-field MRI	*P*
Prevalence	65 (72.2)	54 (75.0)			0.363*
Total number of lesions	322	314	4 (1-16)	5 (1-14)	0.076*
Location of lesions:					
basal ganglia	51 (56.7)	45 (62.5)	2 (0-9)	2 (0-8)	0.160^†^
brainstem	27 (30.0)	18 (25.0)	0 (0-6)	0 (0-4)	0.554^†^
cerebellum	19 (21.1)	24 (33.3)	0 (0-7)	0 (0-6)	0.738^†^

*t test.

†χ^2^ test.

### The association of T2-hyperintensities and age

**Figure 2:**
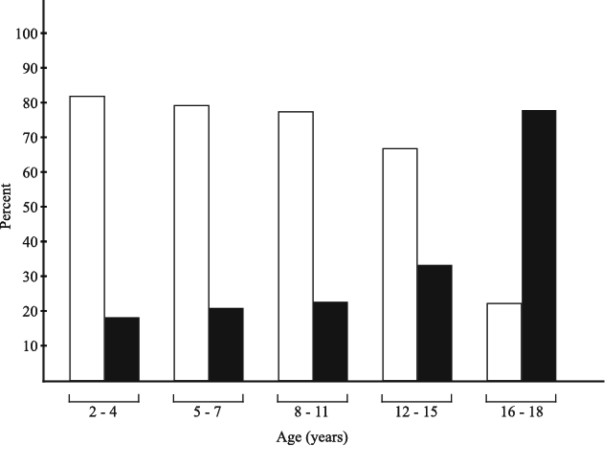
Prevalence of T2-hyperintensities on brain magnetic resonance imaging (MRI) in different age groups of children with neurofibromatosis type 1. The frequency of T2-hyperintensities significantly decreased from age 2-4 years to age 16-18 years (χ^2^ = 12.03, *P* = 0.002). Open bars – T2-hyperintensities present; closed bars – T2-hyperintensities absent.

**Figure 3:**
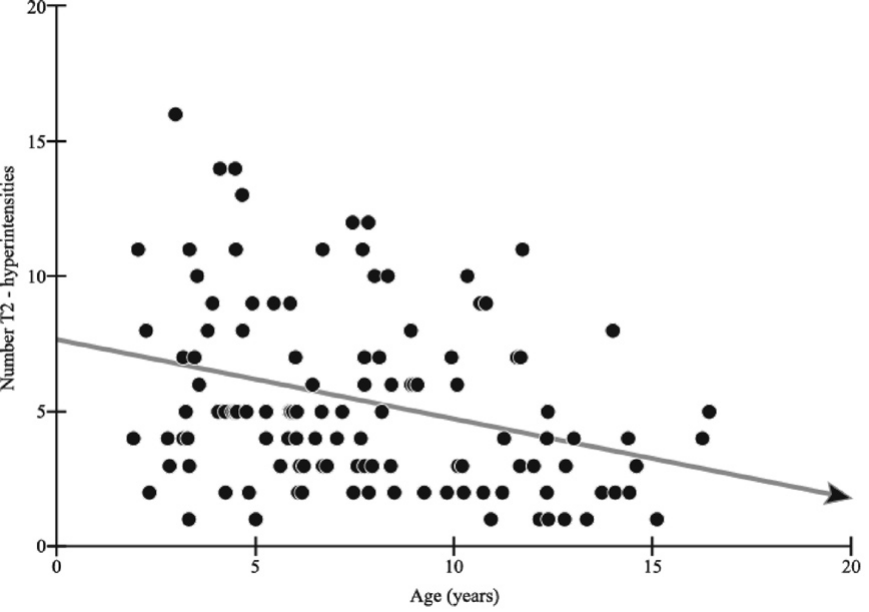
The correlation of the number of T2-hyperintensities and age in 119 children with neurofibromatosis type 1. Pearson correlation showed a significant correlation between the number T2-hyperintensities and age of patients with NF1 (r^2^ linear = 0.100, *P* = 0.011).

There was a significant decrease in the frequency of T2-hyperintensities with age, from 82% at 2-4 years and 79% at 5-7 years to 22% at 16-18 years (*P* = 0.002) (Figure 2). The T2-hyperintensities presence/absence ratio until the end of puberty (age 15) was in favor of the presence. However, in adolescence the presence of T2 hyperintensities significantly decreased and the ratio was in favor of the absence (Figure 2). There was a significant decrease in number of lesions from the earliest age, 2-7 years (total number 368, median 6, range 0-16) until puberty/adolescence, 13-18 years (total number 40, median 3, range 0-8, *P* = 0.011) (Figure 3).

### Clinical diagnostic sensitivity and specificity of presence of T2-hyperintensivities on brain MRI

Clinical diagnostic sensitivity and specificity of the presence of T2-hyperintensities in the group of children with NF1 aged 2-18 years was 74% (95% CI 66%-80.1%) and 98% (95% CI 94.7%-99.6%), respectively, with positive predictive value, negative predictive value, and accuracy rate of 98% (95% CI 93%-99.5%), 79% (95% CI 72.6%-84.2%), and 86% (95% CI 82%-89.5%), respectively. Positive likelihood ratio, negative likelihood ratio, and odds ratio were 40 (95% CI 13-123), 3.7 (95% CI 2.9-4,8), and 1.5 (95% CI 0.4-4.9), respectively.

**Figure 4:**
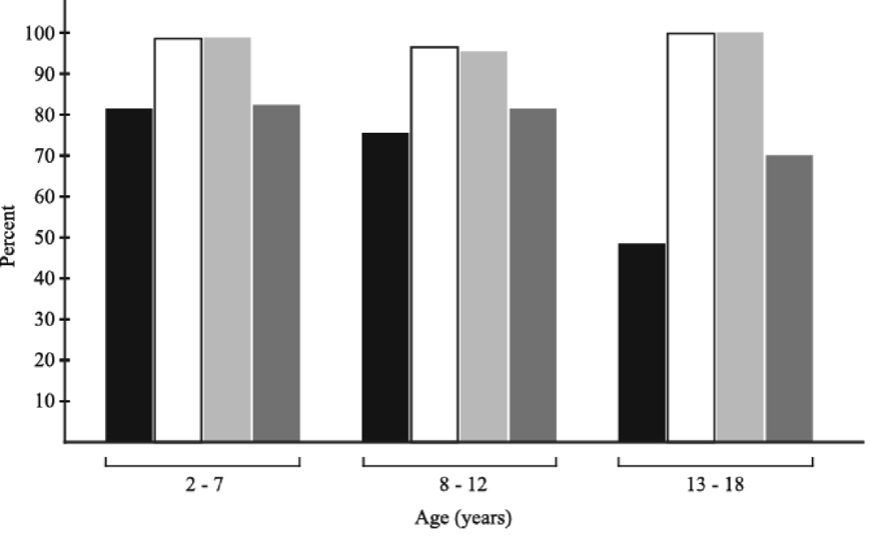
Clinical diagnostic sensitivity, specificity, and positive and negative predictive value of T2-hyperintensities in patients with neurofibromatosis type 1 of different age. Closed bars – sensitivity; open bars – specificity; light gray – positive predictive value; dark gray – negative predictive value.

Sensitivity of T2-hyperintensities (81%, 95% CI 71%-89.1%), positive predictive value (99%, 95% CI 91.8%-99.9%), negative predictive value (82%, 95% CI 72.3%-89.7%), positive likelihood ratio (56.9, 95% CI 8-399), negative likelihood ratio (5.3, 95% CI 3-8), odds ratio (3, 95% CI 0.4-23.3), and the accuracy rate (89%) were highest in the youngest patient group, 2-7 years, and decreased as patients got older. In the age groups 8-12 years and 13-18 years, sensitivity of T2-hyperintensities was 76% (95% CI 62%-86%) and 48% (CI 30%-68%), respectively. Specificity of T2-hyperintensities was high in all age groups, ranging from 97% (95% CI 88%-100%) to 100% (Figure 4).

### ROC analysis

**Figure 5:**
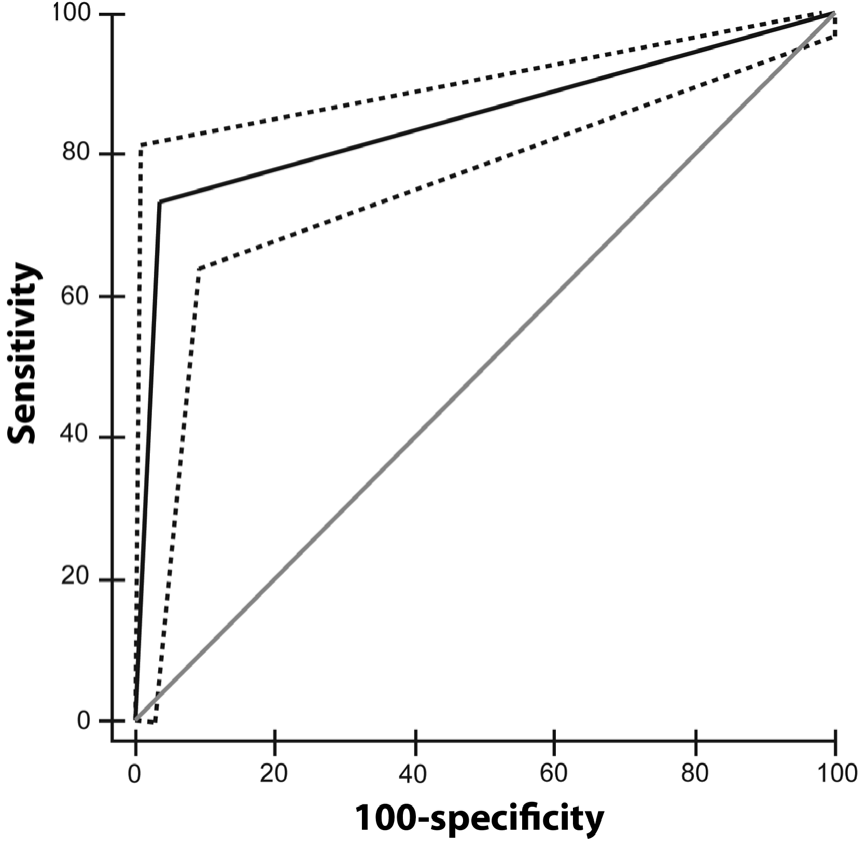
Receiver operating characteristic (ROC) curve of T2-hyperintensities on brain magnetic resonance imaging. ROC curve (full line) and confidence interval (dashed line) for T2-hyperintensities. Gray line defines the area of 0.5.

ROC analysis for the evaluation of diagnostic accuracy in the total group of children (N = 325) showed that multiple T2-hyperintensities on brain MRI can have excellent diagnostic accuracy, with the area under the ROC curve of 0.849 (95% CI 0.805-0.886; *P* < 0.001) (Figure 5).

## Discussion

Our study showed very high specificity of the presence of T2-hyperintensities on brain MRI in diagnosis of NF1 in children – 98% (from 97% to 100% depending on age). The sensitivity was also high – 74% for all age groups, but was the highest in children of early age, until 7 years – 81%. None of the previous studies examined the indicators of diagnostic accuracy of T2-hyperintensities, particularly in the early age. ROC analysis showed an excellent diagnostic accuracy of T2-hyperintensities on brain MRI in children and suggested their clinical usefulness as a diagnostic criterion for NF1, especially in the early age when the definitive clinical diagnosis cannot be established using the NIH diagnostic criteria in all patients. T2-hyperintensities can be used to establish the definitive clinical diagnosis of NF1 as a second, crucial criterion, as it was shown in our 10 patients with the sporadic form of the disease. The finding of multiple T2-hyperintensities in the basal ganglia, brain stem, and cerebellum should raise the suspicion to NF1 and necessitate clinical search for other NF1 features of diagnostic value.

The diagnostic specificity and sensitivity of T2-hyperintensities were examined in only two studies so far (15,17), with results similar to ours. The retrospective study by DeBella et al (15) reported the specificity of T2-hyperintensities of 84% and 74%, respectively, assessed independently by two neuroradiologists in 19 affected children and 19 age-matched controls, aged 4-10. The sensitivity of T2-hyperintensities in the same study was 95% and 100%, respectively. The prospective study by Lopes Ferraz Filho et al (17) showed the specificity of 100% and the sensitivity of 70% in 40 children with NF1 and 48 children from the control group aged 2-18.

The data collection in this study reflects our routine clinical practice. We also recommend routine application of brain MRI in patients with NF1 in the basic clinical evaluation of the severity of disease because of numerous and, sometimes, severe organic and functional changes of central and peripheral nervous system in NF1 (7).

Application of MRI should not be limited only to symptomatic cases with NF1 as it has been recommended so far by some authors (14). We recommend the evaluation by brain MRI in children with NF1 during early childhood, after the second year, before the eighth year, or at the time of establishing the definitive clinical diagnosis if it is established after the previously mentioned age. Brain MRI can confirm the presence of T2-hyperintensities that, apart from their diagnostic value, can contribute to NF1 cognitive impairments through thalamo-cortical dysfunction (18). The recent studies have suggested that T2-hyperintesities may represent pathological foci of hyperplastic or dysplastic glial proliferation with vacuolar or spongiotic changes, with fluid-filled, coalescted, or conflated vacuoles (19). MRI reveals also other specific changes as optic glioma and other brain tumors, which develop in NF1 patients with higher frequency than in general population (20). Other NIH diagnostic changes, as sphenoid dysplasia, also demand neuroradiologic evaluation. Early detection of optic glioma and other neoplasms by MRI in NF1, which can be asymptomatic for longer period, can assure systematic and careful prospective ophthalmologic and neurologic follow-up and prompt intervention in the case of the appearance of symptoms.

The results of our study showed that the MRI systems of different field strength had no significant influence on the detection of brain T2-hyperintensities. Namely, the differences in prevalence, total number of the brain lesions, and their frequency in various brain regions were not significant between the group of patients examined with low/medium-field systems and those examined with high-field systems.

The total prevalence of multiple (≥2) T2-hyperintensities of 74% on the brain MRI in children with NF1 in our study is concordant to the majority of previous studies done in patients of the same age – from 50% to 93% (13,17,21-27). In our control group, only several patients (4.3%) had T2-hyperintensities and only 3 patients (1.8%) had multiple (≥2) lesions. Although the mode of sample selection for our control group cannot present the ideal reflection of healthy pediatric population, the prevalence of T2-hyperintensities in our study is the same to that of the accidental findings of T2-hyperintensities in healthy children up until the age of 18 established by Kim and al (2.2%) (28). In other studies, the presence of T2-hyperintensities was determined in 8 out of 1000 (0.8%) healthy individuals, 3-83 years old (29).

The multiple T2-hyperintensities in our patients were most frequently localized in the basal ganglia (predominantly in the globus pallidus), brainstem, or cerebellum. These results are concordant to the results of practically every former study (10-13,15-17,24). The locations of multiple T2-hyperintensities in our NF1 patients were different from those in healthy individuals. The majority of lesions in the control group were distributed outside the above mentioned three regions, ie, in the cerebrum, which is concordant to the findings of previous studies (15,29).

The agreement in identification of the number and location of T2-hyperintensities between the two neuroradiologists was 77% and 91.4%, respectively. This inconsistency is caused by the subjectivity of visual interpretation and difficulty in defining the exact number of lesions located in the two neighboring brain regions. Namely, T2-hyperintensities in NF1 can present themselves as discrete or diffuse lesions (10). Discrete lesions have well defined margins that are distinct from normal tissue, while diffuse lesions have poorly defined margins and confluent appearance.

In spite of the time limitation of the cross-sectional study design, a relatively large sample of respondents gives us a possibility to conclude that T2-hyperintensities on brain MRI in pediatric patients with NF1 tend to develop in toddlerhood and at preschool age. This is earlier than most NIH diagnostic features, with the exception of multiple *café au lait* spots. The latter feature is in the most cases the first suspected sign with predictive value in the diagnosis of NF1 (30).

This study found a significant association between T2- hyperintensities and age. Both the prevalence and the number of lesions decreased with age, concordant to the findings of other researchers (10,12). Other prospective studies showed that most T2-hyperintensities on brain MRI completely disappeared during late childhood and seemed to be benign (21,24,31,32). On the other hand, cross-sectional study by Szudek and Friedman (16), designed similarly as ours, showed that the frequency of T2-hyperintensities did not change with age.

Our clinical experience and data from this study also show that the clinical diagnosis of NF1 cannot be made using the NIH diagnostic criteria in all children, especially in patients of early age and in sporadic cases. In today’s practice, there are still many cases of late clinical diagnosis, despite of clearly defined clinical diagnostic criteria for NF1 (6). About 50% of children and 33% of adults had been treated for complications associated with NF1 before the clinical diagnosis of the disease was established. Moreover, 35% of children at the age of 5 were not diagnosed. Parents of children suspected of having NF1 want to have the diagnosis confirmed as early as possible (33). A serious diagnostic problem occurs in young children who already develop complications of the disease before showing any of the NIH diagnostic criteria (34). Ninety-seven percent of our patients were diagnosed by using a set of NIH diagnostic criteria until the age of 8, when the *NF1* gene penetration should be complete. The remaining 3% were diagnosed later in life – until the age of 13. The results are similar to the results of previous investigations (8,9).

As far as 15 years ago, several authors (21-26) suggested, due to the impossibility to establish the timely diagnosis of NF1 using NIH diagnostic criteria in some children, especially in their early age, that T2-hyperintensities on the brain MRI were specific and characteristic sign of NF1 and can be an additional diagnostic criterion for the disease. However, the expert consensus did not support the use of T2- hyperintensities because data on their specificity were scarce (14). On the other hand, more recent research confirms the value of T2-hyperintensities as a diagnostic sign for NF1 in children (15,17).

In conclusion, our results of high prevalence, characteristic location, and high specificity and sensitivity of T2-hyperintensities on brain MRI strongly suggest their inclusion as diagnostic criterion for NF1 in children. As a diagnostic sign, they show very high levels of accuracy, the highest until 8 years, when the clinical penetration of the *NF1* gene is not yet finished.
